# Eicosanoid Profiles in the Vitreous Humor of Patients with Proliferative Diabetic Retinopathy

**DOI:** 10.3390/ijms21207451

**Published:** 2020-10-09

**Authors:** Albert L Lin, Richard J Roman, Kathleen A Regan, Charlotte A Bolch, Ching-Jygh Chen, Siva S.R. Iyer

**Affiliations:** 1 Department of Ophthalmology, University of Mississippi School of Medicine, Jackson, MS 39216, USA; alin@umc.edu (A.L.L.); cchen@umc.edu (C.-J.C.); 2 Department of Pharmacology and Toxicology, University of Mississippi School of Medicine, Jackson, MS 39216, USA; rroman@umc.edu; 3 Department of Ophthalmology and Visual Sciences, University of Wisconsin School of Medicine and Public Health, Madison, WI 53726, USA; kregan3@wisc.edu; 4 Department of Ophthalmology, University of Florida College of Medicine, Gainesville, FL 32610, USA; cbolch21@gmail.com

**Keywords:** vitreous, eicosanoids, proliferative diabetic retinopathy, tractional retinal detachment

## Abstract

Proliferative diabetic retinopathy is a potentially blinding sequela of uncontrolled diabetes that involves a complex interaction of pro-angiogenic and inflammatory pathways. In this study, we compared the levels of pro-angiogenic arachidonic acid-derived mediators in human vitreous humor obtained from eyes with high-risk proliferative diabetic retinopathy versus controls. The results indicated that lipoxygenase and cytochrome P450-derived eicosanoids were elevated (5-HETE, 12-HETE, 20-HETE, and 20-COOH-AA)**,** and there appeared to be no differences in levels measured in eyes with tractional retinal detachments versus those without. These results provide further insight into the pathogenesis of this disease and for the development of future potential therapeutic agents that target arachidonic acid metabolites to treat diabetic retinopathy.

## 1. Introduction

Proliferative diabetic retinopathy (PDR) is a sight-threatening condition faced by many diabetic patients [1,2,3,4,5]. On the clinical spectrum of diabetic retinopathy, PDR is considered the advanced form of diabetic retinopathy, heralded by the formation of abnormal retinal neovascularization due to severe retinal ischemia. As a consequence of pathology at the vitreoretinal interface, these patients are at particular risk for permanent retinal damage without treatment [3,4]. Currently, there are multiple treatment options for PDR, of which the gold standard remains pan-retinal photocoagulation (PRP) [6].

In some cases, due to either poor visibility of the fundus or advanced disease, some patients cannot be adequately treated with PRP alone and must undergo pars plana vitrectomy [7]. During this surgery, the vitreous humor and its related posterior cortical attachments are removed, and pan-retinal photocoagulation is applied to the retina via an endolaser probe or indirect laser ophthalmoscopy. This surgery is commonly performed for non-clearing vitreous hemorrhages causing persistent vision loss. In very advanced disease, proliferative diabetic retinopathy can lead to tractional retinal detachments that often require extensive and complex surgical maneuvers for surgical success [8,9]. The visual prognosis for these patients can be quite poor despite successful surgical outcomes.

Currently, vascular endothelial growth factor (VEGF) is the primary target of pharmacologic intervention for PDR. Prior research has confirmed elevations of VEGF in vitreous samples in diabetic retinopathy [10], with VEGF increasing retinal endothelial permeability [11]. Targeting VEGF has been the paradigm for treating diabetic retinopathy in current clinical therapy and is considered the standard of care for diabetic macular edema involving the central macula. More recent clinical research has demonstrated that treating proliferative diabetic retinopathy patients with intravitreal ranibizumab, an anti-VEGF monoclonal-antibody fragment, is not inferior to PRP [12]. Other than steroid treatment, drugs targeting non-VEGF pathways of diabetic retinopathy have yet to enter clinical practice.

**Figure 1:**
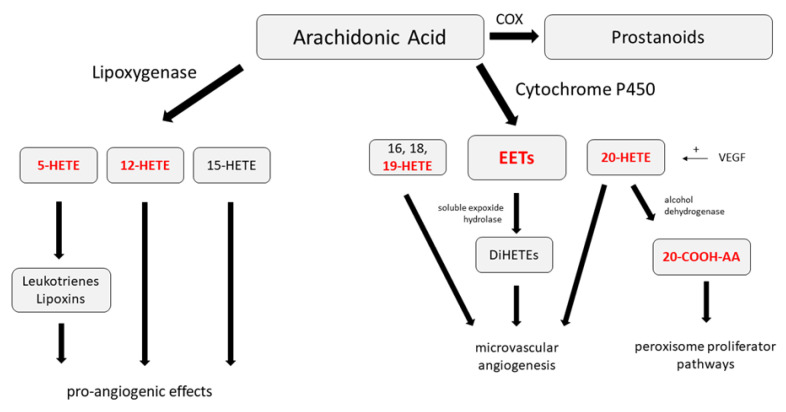
A schematic of arachidonic acid’s hydroxyeicosatetraenoic (HETE), and epoxyeicosatrienoic acid (EET) derivatives and their relationships. HETEs are created by both the lipoxygenase (5-HETE, 12-HETE, 15-HETE) and cytochrome P450 (16-HETE, 18-HETE, 19-HETE, 20-HETE) pathways with dihydroxyeicosatraenoic acid (DiHETE) derived from EET by soluble expoxide hydrolase. 20-carboxy-arachidonic acid (20-COOH-AA) is a metabolite of 20-HETE. Eicosanoids that were significantly different in the vitreous of control and PDR eyes in the current study are shown in bold font COX = cyclooxygenase. HETEs and their derivatives have a role in pro-angiogenic processes [17,18,19,20,21,22,23,24,25,26,27,28].

The current view concerning the pathogenesis of PDR involves an intraocular impetus to form de-novo aberrant retinal vessels to counteract microvascular ischemia [9,13]. This impetus consists of the action of the well-established vascular endothelial growth factor (VEGF), and other pro-angiogenic and pro-fibrotic growth factors (e.g., connective tissue growth factor) [14,15,16]. Arachidonic acid (AA) derivatives (specifically, eicosanoids such as prostaglandins, prostacyclins, hydroxyeicosatetraenoic acid (HETEs), dihydroxyeicosatraenoic acid (DiHETEs), leukotrienes, and epoxyeicosatrienoic acid (EETs) have garnered interest, and have been implicated in retinal pro-angiogenesis. A summary of the three pathways for the metabolism of arachidonic acid and the products formed is presented in Figure 1. Lipoxygenase (LOX) pathways catalyze the hydroxylation of arachidonic acid, and 5-HETE, 12-HETE, and 15-HETE are the primary products of this activity (Figure 1) [17]. Increased levels of 12-LOX have been observed in a mouse model of diabetes [17] and in the retina of a mouse model for retinopathy of prematurity (ROP), which is also characterized by neovascularization of the retina. Further, pharmacologic inhibition or 12-LOX deletion lowered VEGF levels and decreased retinal neovascularization [17].

15-HETE has been reported to be elevated in retinal samples of a mouse model of diabetes and ROP [17]. 15-HETE levels increase under hypoxic conditions [18,19,20], upregulate the expression of VEGF [21,22], and have pro-angiogenic effects that can be blocked by anti-VEGF antibodies [18,19,20,21,29]. Placenta growth factor has also been implicated in triggering the 15-HETE angiogenesis pathway [30]. 15-HETE has also been detected in epiretinal membranes of patients with PDR and with proliferative vitreoretinopathy [31]. Similarly, 12-HETE levels have been shown to be elevated in the human vitreous with PDR compared with nondiabetic controls, and incubation of retinal Muller cells and astrocytes with 12-HETE increases the production of VEGF [17]. Intravitreal 12-HETE induces retinal neovascularization and vascular leakage in murine models, demonstrated by fluorescein angiography [32]. As poorly controlled diabetes can also lead to nephropathy, urinary analysis has revealed that 12-HETE levels are elevated in diabetic patients regardless of plasma creatinine levels, or the degree of proteinuria [33]. Both 12-HETE and 15-HETE increase the permeability of murine retinal endothelial cells, a phenomenon reversed by anti-VEGF treatment [11]. In human vitreous, 5-HETE was also elevated in diabetic patients, especially in those with non-proliferative diabetic retinopathy and in a mouse model of ROP [17].

Cytochrome P450 enzymes lead to the formation of 18-19-, and 20-HETE, and stimulates microvascular angiogenesis [23,24,25,26]. VEGF increases 20-HETE production (Figure 1), and blockade of the formation of 20-HETE prevents the angiogenic effects of VEGF [24]. 20-carboxy-arachidonic acid (20-COOH-AA) is a metabolite of 20-HETE formed by the actions of alcohol dehydrogenase. It is a dual activator of the alpha and gamma peroxisome proliferator pathways that has been implicated in mediating vasodilation [27,28].

In the present study, we profiled the changes in the concentrations of potential pro-angiogenic mediators formed from AA in diabetic retinopathy, primarily LOX- and CYP450 derived HETEs, epoxyeicosatrienoic acid (EETs), and dihydroxyeicosatraenoic acid (DiHETEs).

## 2. Results

**Figure 2:**
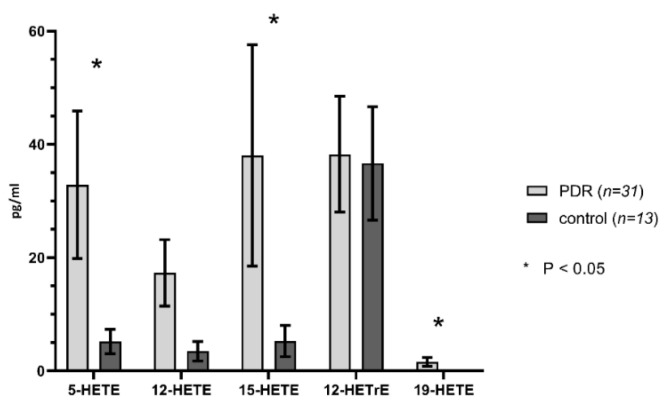
Human vitreous levels of eicosanoids in PDR (*n* = 31) vs. control (*n* = 13) eyes. Comparison of levels (pg/mL) of 5-HETE, 12-HETE, 15-HETE, 12-HETrE, and 19-HETE in PDR vs. controls. * denotes statistical significance (*p* < 0.05).

**Figure 3:**
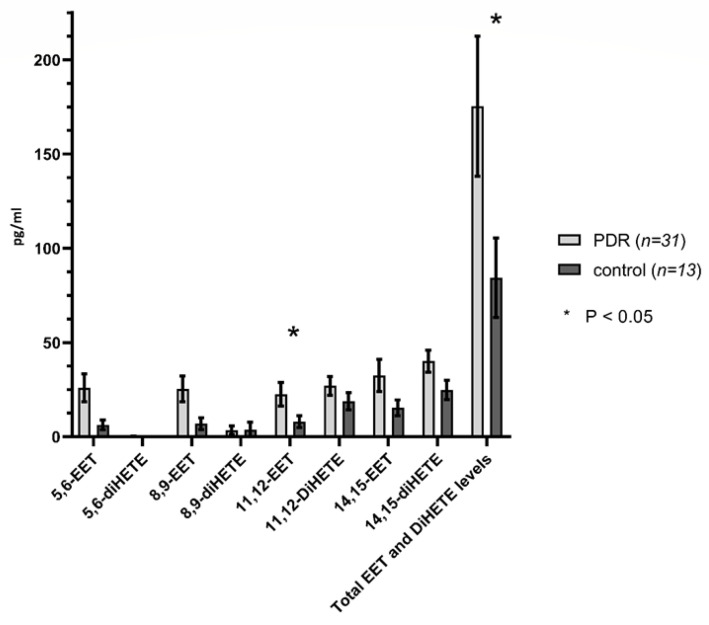
Human vitreous levels of eicosanoids in PDR (*n* = 31) vs. control (*n* = 13) eyes. Comparison of levels (pg/mL) of individual EET and DiHETE, as well as total levels in PDR vs. controls. * denotes statistical significance (*p* < 0.05).

**Figure 4:**
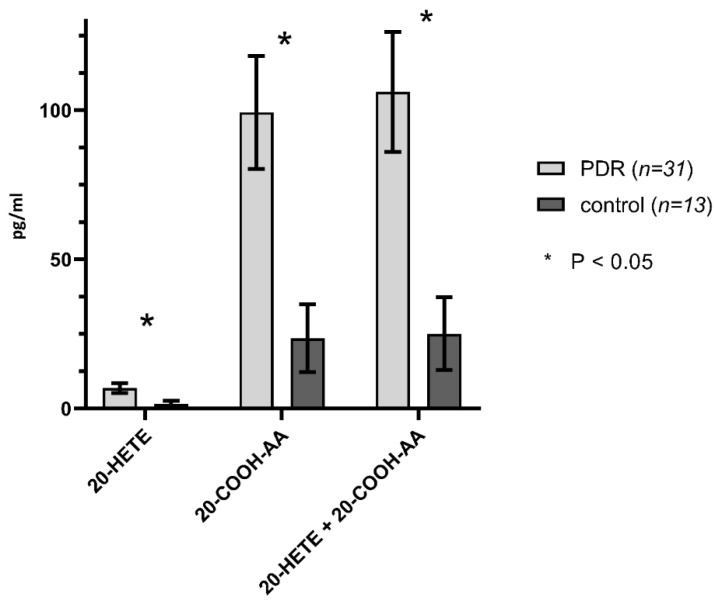
Human vitreous levels of eicosanoids in PDR (*n* = 31) vs. control (*n* = 13) eyes. Comparison of levels (pg/mL) of 20-HETE, 20-COOH-AA in PDR vs. controls. * denotes statistical significance (*p* < 0.05).

A comparison of eicosanoid levels in the vitreous of eyes with proliferative diabetic retinopathy versus controls are presented in Figure 2, Figure 3 and Figure 4 and Table 1. 20-HETE, 12-HETE, 5-HETE, 20-COOH-AA, 11-12-EET levels, and total EET and DiHETE levels, and the sum of the concentrations of 20-HETE and 20-COOH-AA were all significantly higher in PDR eyes compared to control eyes. 20-HETE levels were elevated by 4.5-fold (*p* < 0.01) in PDR eyes than the controls. Similarly, the concentration of 20-COOH-AA (*p* = 0.01) and the sum of the concentrations of 20-HETE and 20-COOH-AA (*p* < 0.01) were 4.2-fold greater in PDR than control eyes. The concentrations of 12-HETE (*p* < 0.03) and 5-HETE (*p* < 0.04) were 5.0 and 6.3-fold higher, respectively, in PDR than control eyes. 19-HETE (*p* < 0.05) levels were undetectable in the control samples and rose to 1.57 ± 0.77 pg/mL in the PDR samples. 11,12-EET levels (*p* < 0.04) were 2.8 times higher in PDR than control eyes, and the sum of EETs and diHETEs concentrations (*p* < 0.04) derived from epoxygenase activity were 2.1 times higher in PDR than control eyes. In contrast, the levels of the lipoxygenase derived 8-, 16- and 18-HETEs, 8,9- and 14,15-EETs, 8,9-, 11,12- and 14,15- DiHETEs and 12R-HETrE were not significantly different in control and PDR eyes.

**Figure 5:**
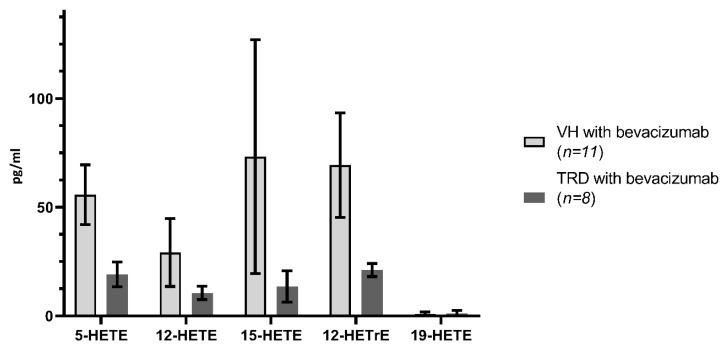
Human vitreous levels of eicosanoids in PDR subtypes treated with bevacizumab, VH (*n* = 11) vs. TRD (*n* = 8) eyes. Comparison of levels (pg/mL) of 5-HETE, 12-HETE, 15-HETE, 12-HETrE, and 19-HETE in VH vs. TRD.

**Figure 6:**
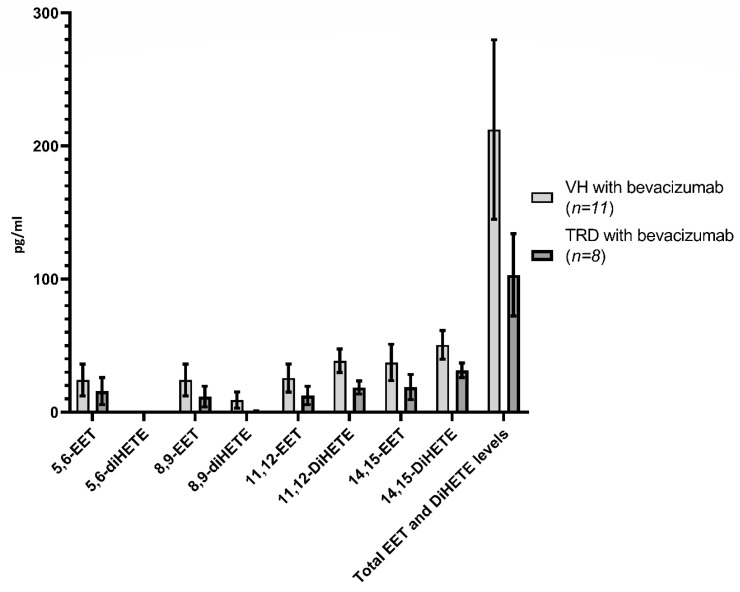
Human vitreous levels of eicosanoids in PDR subtypes treated with bevacizumab, VH (*n* = 11) vs. TRD (*n* = 8) eyes. Comparison of levels (pg/mL) of individual EET and DiHETE as well as total levels in VH vs. TRD.

**Figure 7:**
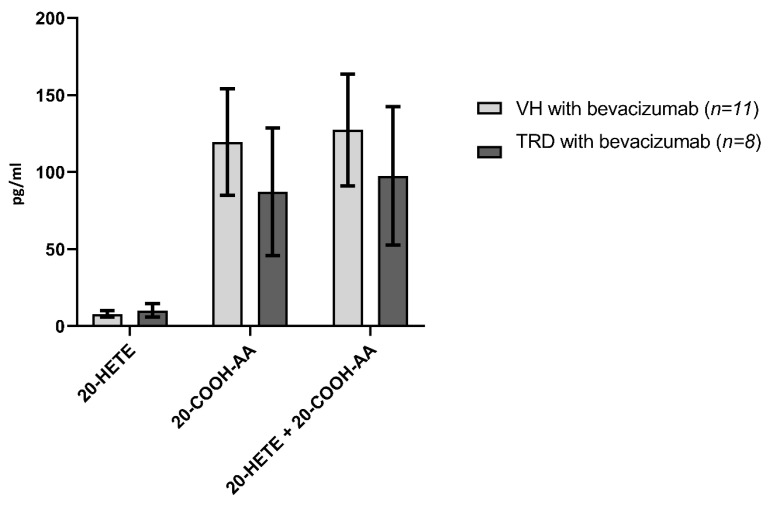
Human vitreous levels of eicosanoids in PDR subtypes treated with bevacizumab, VH (*n* = 11) vs. TRD (*n* = 8) eyes. Comparison of levels (pg/mL) of 20-HETE, 20-COOH-AA in VH vs. TRD.

A comparison of eicosanoid levels in eyes with and without tractional retinal detachments are presented in Figure 5, Figure 6 and Figure 7 and Table 2. The levels of all the eicosanoids studied were not significantly different in the vitreous of eyes with and without TRD.

## 3. Discussion

Anti-VEGF antibodies are now used routinely in the clinical treatment of diabetic retinopathy. Despite the effectiveness of these medications, refractory diabetic macular edema, vitreous hemorrhage, or progression to tractional retinal detachments may still occur. Prior to the advent of anti-VEGF therapy, diabetic retinopathy was commonly treated with corticosteroid-based medications, which act in part by inhibiting phospholipase A2 and the formation of eicosanoids [34]. Diabetic complications not adequately treated with anti-VEGF drugs are often medically treated with pan-retinal photocoagulation (PRP) or intravitreal corticosteroids. This suggests that the angiogenic sequelae causing complications in diabetic retinopathy are not strictly due to the production of VEGF, and other pro-angiogenic inflammatory mediators may also play a role in the progression of diabetic retinopathy. Moreover, there is considerable evidence that pro-angiogenic eicosanoids stimulate the production and release of VEGF and that 20-HETE may be a downstream mediator of the effects of VEGF [35].

For the present analysis, the lipoxygenase and CYP450-derived eicosanoids were profiled due to previous literature supporting a role for these metabolites in angiogenesis in the eyes of diabetic retinopathy [10]. The levels of 5-HETE, 12-HETE, 20-HETE, and 20-COOH-AA in eyes with proliferative diabetic retinopathy (regardless of bevacizumab treatment) were significantly higher than that measured in control eyes (Figure 1, Figure 2, Figure 3 and Figure 4 and Table 1). Schwartzman et al. previously reported that the levels of the lipoxygenase derived eicosanoid, 5-HETE, was significantly higher in the vitreous humor of diabetic eyes [10]. Though 5-HETE itself does not have powerful inflammatory effects (other than stimulating the generation of superoxide in human neutrophils), it is a marker for increased 5-LOX activity. 5-HETE is the precursor for formation of pro-inflammatory leukotrienes, such as leukotriene B4 (LTB4) and the cysteinyl-LTs, and later in the formation of anti-inflammatory mediators (eicosapentaenoic acid (EPA), docosahexaenoic acid (DHA), resolvins, and protectins) involved in the resolution of inflammation. With leukocyte activity being recognized as playing an important role in the development and progression of diabetic retinopathy, the presence of elevated 5-HETE levels in the eyes of these patients imply that activation of the 5-LOX pathway may contribute to the inflammation seen in diabetic retinopathy [36,37]. The clinically beneficial effects of corticosteroids in diabetic retinopathy also indirectly support the activation of this pathway in the progression of diabetic retinopathy. With the availability of anti-leukotriene agents, such as montelukast and ziluteon, this generates an intriguing question as to whether blockade of this pathway could be targeted in translational preclinical research studies and clinical trials as an alternate mechanism for treating diabetic retinopathy.

Guo et al. [35] and Amaral et al. [26] demonstrated that VEGF stimulates the production and release of 20-HETE and that 20-HETE serves as a downstream mediator of the angiogenic effects of VEGF. Indeed, inhibitors of the formation of 20-HETE prevented the angiogenic effect of VEGF applied to the cornea and following electrical stimulation in skeletal muscle [26,35]. Blockade of 20-HETE has also been shown to block the restenosis of carotid arteries following endothelial injury [38]. In the sub-analysis of bevacizumab-treated PDR eyes, 20-HETE levels were notably low and were not significantly different between TRD and non-TRD eyes (VH). Given the previous literature regarding the ability of VEGF to stimulate the production of 20-HETE, it is not surprising that blocking the VEGF receptor with a therapeutic antibody might be expected to decrease levels of 20-HETE. Additional work, however, is needed with larger sample sizes to better understand the relationships between 20-HETE and VEGF production and their interactions in the eyes of patients with PDR.

11-,12 EET and the sum of EET and DiHETE levels were statistically elevated in PDR patients compared to controls. Neither were statistically higher in bevacizumab-treated eyes with tractional detachment versus those without tractional detachment (vitreous hemorrhage). Schwartzman et al. also reported that the levels of EETs are higher in the vitreous humor of nondiabetic control subjects compared to those with diabetic retinopathy [10]. EETs have been associated with anti-inflammatory, cytoprotective, and neuroprotective qualities. However, they also have marked pro-angiogenic effects in the retina via hypoxic stimulation of astrocytes. Otherwise, the role of 11,12-EET in the pathogenesis in diabetic retinopathy is relatively unknown. The present study provides a firm scientific premise for the further exploration of their potential role as epoxygenase metabolites of AA in diabetic retinopathy.

Limitations of the study include a low sample size in regard to the evaluation of the effects of bevacizumab, although our study represents the first and largest study of eicosanoid profiles in human vitreous samples with PDR vs. controls. The lack of a sufficient number of patients with tractional retinal detachments that did not receive bevacizumab prevents a direct evaluation of the effects of bevacizumab in patients with tractional retinal detachments. Despite the stratification of patients with proliferative diabetic retinopathy to those with and without tractional retinal detachment as a means of separating severity, the actual severity of diabetic retinopathy in patients can still differ over a broad-spectrum. It is also possible that systemic diabetic treatment may have unknown effects with measured metabolites in our study. How this might influence the concentration of LOX and CYP-derived metabolites of arachidonic acid is still unknown and may play a part in the varying levels of these metabolites found in the vitreous humor. For example, some diabetic eyes that underwent vitrectomy may have already been treated with PRP or systemic medication, which may have altered the existing levels of lipoxygenase-derived eicosanoids regardless of bevacizumab treatment. The decision of whether to treat an eye with bevacizumab prior to surgery is primarily a surgeon preference (SSRI, CJ); intravitreal administration of bevacizumab prior to surgery decreases intra-operative bleeding and makes surgical dissection of tractional membranes less difficult [9]. Thus, pre-treatment of diabetic tractional retinal detachments can help improve surgical outcomes, especially with very complex tractional detachments [9,39]. This is the primary reason why so few samples were not treated with bevacizumab prior to surgery were available for this study. In our study, no significant differences were found between bevacizumab-treated eyes with VH versus TRD (Figure 5, Figure 6 and Figure 7, Table 2). However, definitive conclusions regarding the effects of bevacizumab on eicosanoid profiles in PDR cannot be made from the current study given the limited number of control samples.

In addition, vitreous humor samples were obtained within seven days of bevacizumab administration. Eicosanoid levels likely would have differed if these samples were collected earlier since eicosanoids have a limited half-life and are rapidly degraded and reincorporated into membrane phospholipid pools. The concentration measured at any particular time represents a snapshot of the balance between the synthesis, breakdown, and reincorporation of these products. Given that the clinical effects of bevacizumab can be seen in a few days, it is reasonable to consider that the effect of bevacizumab on some eicosanoid levels that resolves in less than a week may contribute to the improved clinical appearance of the retina a few days following treatment.

In vivo and ex vivo, bevacizumab and other anti-VEGF inhibitors demonstrate regression of diabetic retinopathy sequelae, such as neovascularization, retinal non-perfusion, and diabetic macular edema. Based on our current understanding of arachidonic acid metabolites in the pathogenesis of diabetic retinopathy, eicosanoid, and phospholipase inhibitors can block VEGF effects in a variety of systems [38]. Thus, despite the higher levels of eicosanoids in eyes with PDR, alternative therapies in the future that target these products may have a powerful positive effect on the severity of diabetic retinopathy.

In summary, this study supports previous findings that lipoxygenase- and CYP-derived eicosanoids are elevated and potentially play a part in the pathogenesis of diabetic retinopathy, specifically 5-HETE, 12-HETE, 20-HETE, and 20-COOH-AA. Eicosanoid profiles between tractional retinal detachment eyes and non-tractional retinal detachment eyes did not show significant differences. The effect of bevacizumab on eicosanoid levels requires further investigation with larger sample sizes. Even with anti-VEGF administration, lipid-based inflammatory markers are still elevated in human vitreous, which could lead to future targeting of these pathways in the treatment of diabetic retinopathy.

## 4. Materials and Methods

This study was approved by the Institutional Review Board of the University of Mississippi and was assigned protocol number 20150028.

### 4.1. Inclusion Criteria

Adult patients that required pars plana vitrectomy for any medically necessary reason and agreed to IRB approved informed consent in accordance with the Tenets of the Declaration of Helsinki were included.

### 4.2. Exclusion Criteria

Any patient that had a prior vitrectomy was excluded. Any patient that had prior intravitreal treatments with any medication within three months (other than bevacizumab within seven days) of surgery were excluded. Vulnerable populations, including those of trauma, children, and geriatric patients (age > 80), were not included in this study.

### 4.3. Sample Collection

At the beginning of vitrectomy for patients with proliferative diabetic retinopathy, 0.7 mL of a pure sample of vitreous humor was obtained with a vitrectomy suction cutter (Constellation, Alcon, Fort Worth, TX, USA) and stored at −80 °C until they were analyzed by LC/MS/MS. Control samples were obtained in a similar fashion from patients who did not have diabetic retinopathy. The vitreous samples of PDR eyes that had received preoperative anti-VEGF therapy (1.25 mg/0.05 mL bevacizumab) 2 to 7 days prior to surgery as part of their treatment were sub-analyzed to compare those that had diabetic tractional retinal detachments and those that did not have diabetic tractional retinal detachments (see data analysis). The quality of sample collection, transport, and storage was carefully monitored. All samples were required to be pure, undiluted vitreous with immediate placement on dry ice in the operating room. Samples were transported to a −80 freezer within two hours of collection. Samples were de-identified and numbered per IRB protocol. Any samples that did not meet these standards were excluded from the analysis.

### 4.4. LC/MS Eicosanoid Assay

Aliquots of the frozen vitreous samples were added to 2 mL of saline acidified with formic acid to pH 3.5. The samples were extracted twice with 3 mL of ethyl acetate after the addition of 2 ng of a d6-20-HETE internal standard. This organic extraction isolates free fatty acids from proteins and phospholipids. The organic phase was washed with 1 mL of H_2_O to remove residual acid, and the organic phase was collected and dried under nitrogen. The samples were reconstituted with 50% methanol in water, and the metabolites of arachidonic acid were separated using a Dionex (Sunnyvale, CA, USA) HPLC system and an ABsciex 4000 Q-trap tandem mass spectrometer with electrospray ionization (ABsciex, Foster City, CA, USA) as previously described [25,38,40]. Separation of the metabolites formed was achieved by HPLC using a reverse-phase column (Beta Basic C18, 150 × 2.1 mm, 3 um; Thermo Hypersil-Keystone, Bellefonte, PA, USA), at a flow rate of 300 L/min. The column was equilibrated with 66.7% of a mobile phase A containing water/acetonitrile/methanol/acetic acid (90/8.5/1.4/0.1, v/v/v/v), and 33.3% mobile phase B, acetonitrile/methanol/acetic acid (85/15/0.1 v/v/v) for 5 min following injection of the sample. The percentage of mobile phase B was ramped to 53.5% over a 10 min period and held for 5 minutes, then ramped to 94.4% mobile phase B over 7 min and held there for 5 min. We have previously documented that this HPLC protocol is sufficient to provide unique baseline separation of all the EETs, DiHETEs, and HETE metabolites [40].

The products were ionized in the negative ion mode and analyzed using multiple reaction monitoring (MRM) with the following instrument settings: Electrospray voltage −4500 volts, curtain gas 30, gas 1–50, temperature 600 °C, gas 2–50, and unit resolution. The following transitions were monitored for each metabolite of arachidonic acid; m/z 337-207 (14,15-DiHETE), m/z 337-167 (11,12-DiHETE), m/z 337-127 (8,9-DiHETE), m/z 319-231 (19-HETE), m/z 319-245 (20-HETE), m/z 319-261 (18-HETE), m/z 337-145 (5,6-DiHETE), m/z 319-233 (16-HETE), m/z 319-175 (15-HETE), m/z 319-149 (11-HETE), m/z 319-179 (12-HETE), m/z 319-155 (8-HETE), m/z 319-203 (5-HETE), m/z 319-175, (14,15-EET), m/z 319-167 (11,12-EET), m/z 319-127 (8,9EET), m/z 319-191 (5,6-EET), and m/z 325-281/307 (d_6_-20-HETE). Standard curves were generated from the ratio of peak area ratios using a range of 0.02 to 20 pg/mL for each of the metabolites versus and 2 ng of d_6_-20-HETE. All of the eicosanoid standards and the d6-20-HETE were purchased from Cayman Chemical (Ann Arbor, MI, USA).

### 4.5. Data Analysis

A total of 58 samples were collected from patients. Any sample that did not meet standards of quality as described above, including retrieval and/or transport and was excluded from the analysis. Blank samples that contained little or no eicosanoids across the board <5 pg/mL for multiple analyses were excluded. If any of the eicosanoids were present in the sample at a concentration >5 pg/mL, then the values for all the analytes were included in the analysis. Overall, 31 PDR samples and 13 control samples were included in the data analysis. Sixteen of the 31 PDR samples were from TRD eyes, and 15 samples were collected from non-TRD eyes (vitreous hemorrhage). A comparison of the values obtained in TRD eyes versus non-TRD eyes (vitreous hemorrhage) was also performed. As half of TRD eyes were treated with bevacizumab (8/16), only those samples that were treated with bevacizumab in both groups (TRD and VH) were compared to each other to better isolate the effect of the disease state on eicosanoid levels rather than the potential influence of bevacizumab. The 13 control vitreous samples were obtained from the eyes of patients that underwent vitrectomy for reasons other than diabetic retinopathy. The control samples included 4 with epiretinal membranes, 5 rhegmatogenous retinal detachments, 2 macular holes, 1 posterior vitreous detachment opacity (floater), and 1 vitreous hemorrhage (macroaneurysm from hypertension).

### 4.6. Statistics

Mean values ± 1 standard error of measurement (SEM) are presented. The significance of differences in mean values of 20-HETE, 12-HETE, 5-HETE, 20-COOH-AA, 12-HETrE, 11,12-EET, 14,15-EET, 11,12-DiHETE, 14,15-DiHETE, EETs and DiHETEs and the sum of the concentration of 20-HETE plus its 20-COOH-AA metabolite derived from CYP450 ω-hydroxylase level in patients with proliferative diabetic retinopathy versus controls were compared using an unpaired t-test (two groups: PDR versus control) with Welch’s correction via statistical software (GraphPad Prism 8.01, GraphPad Software). A *p* value < 0.05 was considered statistically significant.

## Figures and Tables

**Table 1:** Mean levels (pg/mL) +/− standard error of measurement (SEM) in human vitreous of lipoxygenase and cytochrome P450-derived eicosanoids in proliferative diabetic retinopathy (PDR, *n* = 31) versus controls (*n* = 13).

Metabolite	PDR ± SEM (31)	Control ± SEM (13)	*p*-Value
5-HETE	32.85 ± 13.04	5.185 ± 2.155	0.0444
12-HETE	17.32 ± 5.869	3.462 ±1.698	0.0297
15-HETE	38.042 ± 19.526	5.269 ± 2.763	0.1066
19-HETE	1.594 ± 0.7731	0	0.048
20-HETE	6.884 ± 1.671	1.538 ±1.042	0.0096
20-COOH-AA	99.28 ± 18.919	23.56 ±11.302	0.0013
11,12-EET	22.684 ± 6.234	8.052 ± 3.070	0.0367
14,15-EET	32.629 ± 8.507	15.408 ± 4.142	0.0544
11,12-DiHETE	27.037 ± 4.876	18.892 ± 4.515	0.2283
14,15-DiHETE	40.13 ± 5.950	24.831 ± 5.150	0.0594
12-HETrE	38.26 ± 10.23	36.646 ±10.018	0.9108
Total ꞷ-hydroxylase level	106.2 ± 20.118	25.1 ±12.183	0.0013
Total epoxygenase level	175.403 ± 37.245	84.354 ±21.101	0.0394

**Table 2:** Mean levels (pg/mL) +/− standard error of measurement (SEM) of lipoxygenase and cytochrome P450-derived eicosanoids in bevacizumab-treated human vitreous in vitreous hemorrhage (VH, *n* = 11) vs. tractional retinal detachment (TRD, *n* = 8) eyes.

Metabolite	VH ± SEM (11)	TRD ± SEM (8)	*p*-Value
5-HETE	55.772 ± 36.114	19.088 ± 5.708	0.3383
12-HETE	29.164 ± 15.587	10.575 ± 3.046	0.2671
15-HETE	73.273 +/− 53.75	13.575 ± 7.183	0.2959
20-HETE	7.927 ± 2.169	10.213 ± 4.280	0.6436
20-COOH-AA	119.518 ± 34.719	87.338 ± 41.407	0.5603
Total ꞷ-hydroxylase level	127.446 ± 36.211	97.550 ± 44.996	0.6124
12-HETrE	69.355 ± 24.072	21.713 ± 10.290	0.0913
11,12-EET	25.645 ± 10.543	12.563 ± 6.829	0.313
14,15-EET	37.381 ± 13.720	18.838 ± 9.437	0.2815
11,12-DiHETE	38.691 ± 8.852	18.829 ± 5.510	0.0658
14,15-DiHETE	50.582 ± 10.828	31.414 ± 5.510	0.1366
Total epoxygenase level	212.363 ± 67.437	103.200 ± 30.979	0.1638

## References

[1] Mohamed Q., Gillies M.C., Wong T.Y. (2007). Management of diabetic retinopathy: A systematic review. JAMA.

[2] Javitt J.C., Aiello L.P., Chiang Y., Ferris F.L., Canner J.K., Greenfield S. (1994). Preventive eye care in people with diabetes is cost-saving to the federal government. Implications for health-care reform. Diabetes Care.

[3] Klein R., Klein B.E., Moss S.E., Cruickshanks K.J. (1995). The Wisconsin epidemiologic study of diabetic retinopathy. XV. The long-term incidence of macular edema. Ophthalmology.

[4] (1991). Fundus Photographic Risk Factors for Progression of Diabetic Retinopathy: ETDRS Report Number 12. Ophthalmology.

[5] Kempen J.H., O’Colmain B.J., Leske M.C., Haffner S.M., Klein R., Moss S.E., Taylor H.R., Hamman R.F. (2004). The prevalence of diabetic retinopathy among adults in the United States. Arch. Ophthalmol. 1960.

[6] Giuliari G.P. (2012). Diabetic retinopathy: Current and new treatment options. Curr. Diabetes Rev..

[7] Stefánsson E. (2001). The therapeutic effects of retinal laser treatment and vitrectomy. A theory based on oxygen and vascular physiology. Acta Ophthalmol. Scand..

[8] Michels R. (1986). Basic and Advanced Vitreous Surgery.

[9] Iyer S.R., Regan K.A., Burnham J.M., Chen Ching J. (2019). Surgical management of diabetic tractional retinal detachments. Surv. Ophthalmol..

[10] Schwartzman M.L., Iserovich P., Gotlinger K., Bellner L., Dunn M.W., Sartore M., Grazia P.M., Leonardi A., Sathe S., Beaton A. (2010). Profile of lipid and protein autacoids in diabetic vitreous correlates with the progression of diabetic retinopathy. Diabetes.

[11] Othman A., Ahmad S., Megyerdi S., Mussell R., Choksi K., Maddipati K.R., Elmarakby A., Rizk N., Al-Shabrawey M. (2013). 12/15-Lipoxygenase-derived lipid metabolites induce retinal endothelial cell barrier dysfunction: Contribution of NADPH oxidase. PLoS ONE.

[12] Elman M.J., Aiello L.P., Beck R.W., Bressler N.M., Bressler S.B., Edwards A.R., Ferris F.L., Friedman S.M., Glassman A.R., Diabetic Retinopathy Clinical Research Network (2010). Randomized trial evaluating ranibizumab plus prompt or deferred laser or triamcinolone plus prompt laser for diabetic macular edema. Ophthalmology.

[13] Frank R.N. (1991). On the pathogenesis of diabetic retinopathy. A 1990 update. Ophthalmology.

[14] Van Geest R.J., Klaassen I., Lesnik-Oberstein S.Y., Tan H.S., Mura M., Goldschmeding R., Van Noorden C.J., Schlingemann R.O. (2013). Vitreous TIMP-1 levels associate with neovascularization and TGF-β2 levels but not with fibrosis in the clinical course of proliferative diabetic retinopathy. J. Cell Commun. Signal..

[15] Aiello L.P., Avery R.L., Arrigg P.G., Keyt B.A., Jampel H.D., Shah S.T., Pasquale L.R., Thieme H., Iwamoto M.A., Park J.E. (1994). Vascular endothelial growth factor in ocular fluid of patients with diabetic retinopathy and other retinal disorders. N. Engl. J. Med..

[16] Adamis A.P., Miller J.W., Bernal M.T., D’Amico D.J., Folkman J., Yeo T.K., Yeo K.T. (1994). Increased vascular endothelial growth factor levels in the vitreous of eyes with proliferative diabetic retinopathy. Am. J. Ophthalmol..

[17] Al-Shabrawey M., Mussell R., Kahook K., Tawfik A., Eladl M., Sarthy V., Nussbaum J., El-Marakby A., Park S.Y., Gurel Z. (2011). Increased expression and activity of 12-lipoxygenase in oxygen-induced ischemic retinopathy and proliferative diabetic retinopathy: Implications in retinal neovascularization. Diabetes.

[18] Stuart M.J., Walenga R.W., Setty B.N., Phelps D.L. (1990). Effects of changes in oxygen tension on lipoxygenase metabolites. Serum 15-HETE is increased in kittens exposed to hyperoxia. Biol. Neonate..

[19] Bajpai A.K., Blaskova E., Pakala S.B., Zhao T., Glasgow W.C., Penn J.S., Johnson D.A., Rao G.N. (2007). 15(S)-HETE production in human retinal microvascular endothelial cells by hypoxia: Novel role for MEK1 in 15(S)-HETE induced angiogenesis. Invest. Ophthalmol. Vis. Sci..

[20] Bajpai A.K., Blaskova E., Pakala S.B., Zhao T., Glasgow W.C., Penn J.S., Johnson D.A., Rao G.N. (2017). Key role of 15-LO/15-HETE in angiogenesis and functional recovery in later stages of post-stroke mice. Sci. Rep..

[21] Soumya S.J., Binu S., Helen A., Anil Kumar K., Reddanna P., Sudhakaran P.R. (2012). Effect of 15-lipoxygenase metabolites on angiogenesis: 15(S)-HPETE is angiostatic and 15(S)-HETE is angiogenic. Inflamm Res..

[22] Chen L., Zhu Y.-M., Li Y.-N., Li P.-Y., Wang D., Liu Y., Qu Y.-Y., Zhu D.-L., Zhu Y.-L. (2017). The 15-LO-1/15-HETE system promotes angiogenesis by upregulating VEGF in ischemic brains. Neurol. Res..

[23] Chen L., Joseph G., Zhang F.F., Nguyen H., Jiang H., Gotlinger K.H., Falck J.R., Yang J., Schwartzman M.L., Guo A.M. (2016). 20-HETE contributes to ischemia-induced angiogenesis. Vascul. Pharmacol..

[24] Chen L., Ackerman R., Saleh M., Gotlinger K.H., Kessler M., Mendelowitz L.G., Falck J.R., Arbab A.S., Scicli A.G., Schwartzman M.L. (2014). 20-HETE regulates the angiogenic functions of human endothelial progenitor cells and contributes to angiogenesis in vivo. J. Pharm. Exp. Ther..

[25] Fan F., Sun C.W., Maier K.G., Williams J.M., Pabbidi M.R., Didion S.P., Falck J.R., Zhuo J., Roman R.J. (2013). 20-Hydroxyeicosatetraenoic acid contributes to the inhibition of K+ channel activity and vasoconstrictor response to angiotensin II in rat renal microvessels. PLoS ONE.

[26] Amaral S.L., Maier K.G., Schippers D.N., Roman R.J., Greene A.S. (2003). CYP4A metabolites of arachidonic acid and VEGF are mediators of skeletal muscle angiogenesis. Am. J. Physiol. Heart Circ. Physiol..

[27] Fang X., Faraci F.M., Kaduce T.L., Harmon S., Modrick M.L., Hu S., Moore S.A., Falck J.R., Weintraub N.L., Spector A.A. (2006). 20-Hydroxyeicosatetraenoic acid is a potent dilator of mouse basilar artery: Role of cyclooxygenase. Am. J. Physiol. Heart Circ. Physiol..

[28] Fang X., Faraci F.M., Kaduce T.L., Harmon S., Modrick M.L., Hu S., Moore S.A., Falck J.R., Weintraub N.L., Spector A.A. (2004). 20-hydroxyeicosatetraenoic acid (20-HETE) metabolism in coronary endothelial cells. J. Biol. Chem..

[29] Ma C., Li Y., Ma J., Liu Y., Li Q., Niu S., Shen Z., Zhang L., Pan Z., Zhu D. (2011). Key role of 15-lipoxygenase/15-hydroxyeicosatetraenoic acid in pulmonary vascular remodeling and vascular angiogenesis associated with hypoxic pulmonary hypertension. Hypertension Dallas. Tex. 1979.

[30] Ma C., Wang Y., Shen T., Zhang C., Ma J., Zhang L., Liu F., Zhu D. (2013). Placenta growth factor mediates angiogenesis in hypoxic pulmonary hypertension. Prostaglandins Leukot. Essent. Fat. Acids.

[31] Augustin A.J., Grus F.H., Koch F., Spitznas M. (1997). Detection of eicosanoids in epiretinal membranes of patients suffering from proliferative vitreoretinal diseases. Br. J. Ophthalmol..

[32] Ibrahim A.S., Elshafey S., Sellak H., Hussein K.A., El-Sherbiny M., Abdelsaid M., Rizk N., Beasley S., Tawfik A.M., Smith S.B. (2015). A lipidomic screen of hyperglycemia-treated HRECs links 12/15-Lipoxygenase to microvascular dysfunction during diabetic retinopathy via NADPH oxidase. J. Lipid Res..

[33] Antonipillai I., Nadler J., Vu E.J., Bughi S., Natarajan R., Horton R. (1996). A 12-lipoxygenase product, 12-hydroxyeicosatetraenoic acid, is increased in diabetics with incipient and early renal disease. J. Clin. Endocrinol. Metab..

[34] Martidis A., Duker J.S., Greenberg P.B., Rogers A.H., Puliafito C.A., Reichel E., Baumal C. (2002). Intravitreal triamcinolone for refractory diabetic macular edema. Ophthalmology.

[35] Guo A.M., Arbab A.S., Falck J.R., Chen P., Edwards P.A., Roman R.J., Scicli A.G. (2007). Activation of vascular endothelial growth factor through reactive oxygen species mediates 20-hydroxyeicosatetraenoic acid-induced endothelial cell proliferation. J. Pharm. Exp. Ther..

[36] Gubitosi-Klug R.A., Talahalli R., Du Y., Nadler J.L., Kern T.S. (2008). 5-lipoxygenase, but not 12/15-lipoxygenase, contributes to degeneration of retinal capillaries in a mouse model of diabetic retinopathy. Diabetes.

[37] Talahalli R., Zarini S., Sheibani N., Murphy R.C., Gubitosi-Klug R.A. (2010). Increased synthesis of leukotrienes in the mouse model of diabetic retinopathy. Investig. Ophthalmol. Vis. Sci..

[38] Orozco L.D., Liu H., Perkins E., Johnson D.A., Chen B.B., Fan F., Baker R.C., Roman R.J. (2013). 20-Hydroxyeicosatetraenoic acid inhibition attenuates balloon injury-induced neointima formation and vascular remodeling in rat carotid arteries. J. Pharm. Exp. Ther..

[39] Arevalo J.F., Lasave A.F., Kozak I., Al Rashaed S., Al Kahtani E., Maia M., Farah M.E., Cutolo C., Brito M., Osorio C. (2019). Preoperative bevacizumab for tractional retinal detachment in proliferative diabetic retinopathy: A prospective randomized clinical trial. Am. J. Ophthalmol..

[40] Dreisbach A.W., Smith S.V., Kyle P.B., Ramaiah M., Amenuke M., Garrett M.R., Lirette S.T., Griswold M.E., Roman R.J. (2014). Urinary CYP eicosanoid excretion correlates with glomerular filtration in African-Americans with chronic kidney disease. Prostaglandins Other Lipid Mediat..

